# Aberrant expression of five miRNAs in papillary thyroid carcinomas

**DOI:** 10.1002/jcla.23907

**Published:** 2021-07-16

**Authors:** De‐hui Qiao, Xue‐mei He, Xian Deng, Yi‐chi Ji, Hui Yang, Lian Cheng, Xiang‐yu Zhou

**Affiliations:** ^1^ Department of Thyroid Surgery Affiliated Hospital of Southwest Medical University Luzhou China; ^2^ Department of Central Laboratory Affiliated Hospital of Southwest Medical University Luzhou China

**Keywords:** clinicopathological features, diagnosis, miRNA, molecular marker, papillary thyroid carcinoma

## Abstract

**Background:**

The miRNAs play critical roles in the progression of various tumors. Our study aimed to screen and identify miRNAs to investigate their diagnostic and prognostic value for papillary thyroid carcinoma (PTC).

**Methods:**

miRNAs were evaluated in PTC (*n* = 30) tissues, A‐PTC (*n* = 30), benign nodules (*n* = 35) and A‐benign nodules (*n* = 35). The expression levels of five miRNAs were quantified using real‐time, quantitative PCR. ROC analysis was used to evaluate the miRNA diagnostic value.

**Results:**

The expression of miR‐1296‐5p, miR‐1301‐3p, and miR‐532‐5p was significantly downregulated (*p* = 0.0001, *p* = 0.0006, *p* = 0.0024, respectively), while miR‐551b‐3p and miR‐455‐3p were significantly upregulated in PTC tissues compared to A‐PTC tissues (*p* = 0.0005, *p* = 0.0046, respectively). Interestingly, the expression of miR‐1296‐5p was downregulated, while miR‐551b‐3p and miR‐455‐3p were upregulated in the A‐PTC group compared to the A‐benign group. Moreover, the miR‐1296‐5p expression level was associated with tumor size, the number of foci and the TNM stage; the miR‐455‐3p expression level was correlated with patient age, tumor size, and TNM stage; and the miR‐532‐5p expression level was correlated with patient age, lymph node metastasis and TNM stage correspondingly. ROC analysis revealed that the AUCs for miR‐1301‐3p, miR‐1296‐5p, miR‐455‐3p, miR‐532‐5p, and miR‐551b‐3p were 0.773, 0.790, 0.783, 0.744, and 0.650, respectively.

**Conclusions:**

Our results indicated that miR‐1296‐5p, miR‐1301‐3p, miR‐532‐5p, miR‐551b‐3p, and miR‐455‐3p are aberrantly expressed in papillary thyroid carcinomas and correlated with clinicopathological features. ROC curve analysis indicated that these five miRNAs have a potential diagnostic value. Consequently, we speculate that the five altered miRNAs may serve as potential diagnostic and prognostic biomarkers for PTC.

## Graphical abstract

miR‐1296‐5p, miR‐1301‐3p and miR‐532‐5p were significantly downregulated (*p* = 0.0001, *p* = 0.0006, *p* = 0.0024, respectively), while miR‐551b‐3p and miR‐455‐3p were significantly upregulated in PTC tissues compared to A‐PTC (*p* = 0.0005, *p* = 0.0046, respectively). Aberrant expression of miR‐1296‐5p, miR‐532‐5p, and miR‐455‐3p are correlation with clinical‐pathological features. ROC cure indicated that those five miRNAs have diagnostic value.

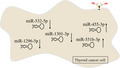

## 1 INTRODUCTION

Thyroid carcinoma is the most common form of endocrine system malignancy, and the incidence of thyroid cancer, especially papillary thyroid cancer (PTC), has increased worldwide over the past several decades.^1^ It is well known that the prognosis of PTC is very good due to its low malignancy and invasiveness. However, approximately 10% of patients still develop distant metastases or die.^2^ Currently, although fine needle aspiration cytology (FNAC) is recognized as the gold standard for PTC diagnosis, there are still some PTCs that cannot be diagnosed by FNAC before surgery. In addition, FNAC is not suitable for estimating malignancy and invasiveness, which usually results in clinical overtreatment. Therefore, identifying suitable markers that can determine whether patients exhibit PTC is a great challenge for clinical diagnosis and crucial for employing the right treatment.^3^

MicroRNAs are endogenous noncoding RNAs, and most studies have revealed that miRNAs are aberrantly expressed in tumors and may play onco‐ or anticancer roles.^4^ There are a large number of abnormal miRNAs that may be associated with PTC. In addition, we do not know the relationship between these abnormal miRNAs and clinical PTC pathological features. This study sought to select some miRNAs to verify clinical PTC cases and analyze their correlation with clinicopathological features. Based on the literature review and the online tool Oncomir (Table S1), among the five selected miRNAs, we found that miR‐1296‐5p, miR‐1301‐3p, miR‐532‐5p, miR‐551b‐3p, and miR‐455‐3p were aberrantly expressed in papillary thyroid carcinomas and correlated with clinicopathological features. ROC curve analysis indicated that the five miRNAs can distinguish PTC cases, benign nodules, and adjacent normal tissues. Therefore, we speculate that these five altered miRNAs may serve as potential diagnostic and prognostic biomarkers for PTC.

## 2 MATERIALS AND METHODS

### 2.1 Thyroid tissue samples

Sixty‐five thyroid samples were obtained from surgical operations conducted in the Department of Thyroid Surgery of the Affiliated Hospital of Southwest Medical University from August 2017 to November 2018. Thirty of the 65 cases were diagnosed with PTC, and 35 were diagnosed with benign nodules by at least two professional pathologists. Patients who had ever received radiotherapy, chemotherapy, or any other preoperative treatment were excluded. Detailed information on the patients is summarized in Table 1. Tumor tissues and adjacent normal tissues (at least 1 cm away from the edge of the tumor) 1 cm from the nodule were collected during the operation, immediately frozen in liquid nitrogen and then stored in a low‐temperature refrigerator at −80°C until use. All samples were divided into four groups, including PTC tissues and corresponding adjacent PTC (A‐PTC) tissues, benign nodule tissues and corresponding adjacent benign nodule (A‐Benign) tissues. This study was approved by the Ethics Committee Board of Southwest Medical University (The NO. is KY2019089), and informed consent was obtained from all patients. The experiments abided by the principles of the Declaration of Helsinki.

**TABLE 1:** Detailed information on the patients

Clinical features	Group	*n*	Clinical features	Group	*n*
Age	≥45	17	ATA classification	Low	18
	<45	13		Mid‐High	12
Sex	Female	19	TNM staging	I	19
	Male	11		Ⅱ﹣Ⅳ	11
Tumor size	≥2 cm	15	Tumor subtype	PTC	26
	<2 cm	15		FVPTC	4
Number of foci	Multi	13	Tg	<77 ng/ml	14
	Single	17		≥77 ng/ml	16
Lymph node metastasis	Yes	20	Extra thyroidal extension	Yes	5
	No	10		No	25

Abbreviations: ATA, American thyroid association guidelines; Tg, thyroglobulin.

### 2.2 RNA Isolation and Relative qPCR

Total RNA was extracted from 50 mg tissues following the manufacturer's instructions (Qiagen, miRNeasy Mini Kit). RNA was quantified with a Nanodrop 2000 (NanoDrop, Thermo Fisher Scientific). Real‐time quantitative PCR (qPCR; Qiagen, SYBR Green PCR kit) was performed in triplicate according to the manufacturer's instructions. The relative expression levels were calculated by the 2^−ΔΔCt^ method with U6 as the reference gene. ATA classification was used to assess the risk of recurrence. The TNM stage was assessed according to the American Joint Committee on Cancer staging systems. All experiments were repeated three times and the average value was taken.

### 2.3 Statistical analysis

SPSS 19.0 (SPSS Inc.) was used to analyze the data. The Kolmogorov‐Smirnov (K‐S) test to test whether the data obtained were in line with normal distribution. An independent sample *t*‐test was used for normally distributed data, and the Mann‐Whitney *U*‐test was applied for abnormally distributed data (Q25, Q75). One‐way analysis of variance (ANOVA) was used to compare the differences among the three groups. A receiver operating characteristic (ROC) curve was used to test the diagnostic utility. The AUC and cut‐off values were analyzed by MedCalc. Differences were considered statistically significant when *p* < 0.05.

## 3 RESULTS

### 3.1 MiRNA expression in PTC, benign nodules, and adjacent normal tissues

**FIGURE 1:**
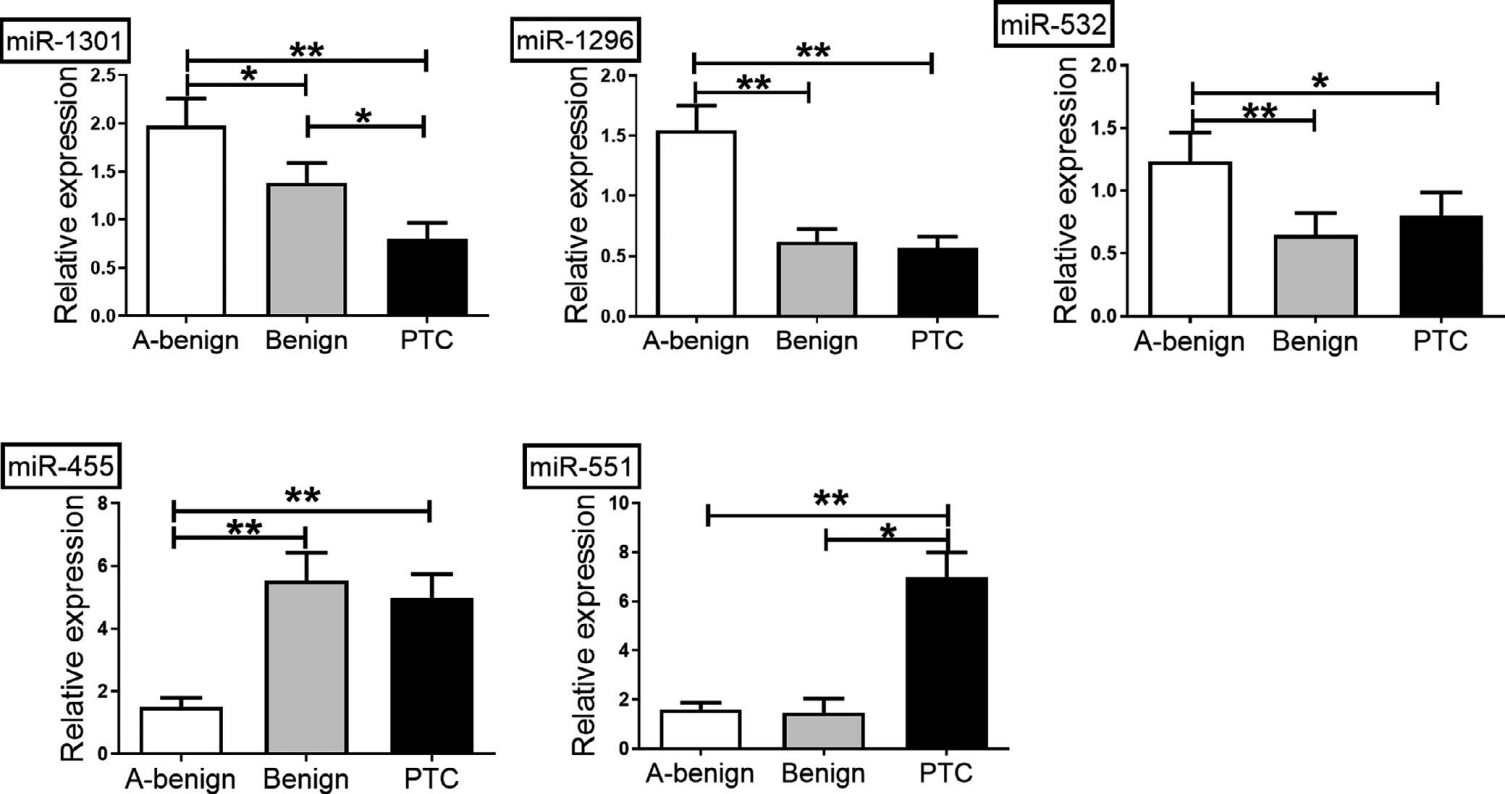
miRNA expression in PTC, benign nodules, and adjacent normal tissues. Compared to adjacent normal tissues, the expression of miR‐1301‐3p, miR‐1296‐5p, and miR‐532‐5p was significantly decreased, while the expression of miR‐455‐3p and miR‐551b‐3p was increased in PTC tissues. Compared to benign nodules, miR‐1301‐3p was downregulated, and miR‐551b‐3p was upregulated in PTC tissues. The *p*‐value was calculated using one‐way analysis of variance (ANOVA). (*p < 0.05, **p < 0.01)

In this study, we measured the expression of five miRNAs in PTC tissues, benign nodules, and adjacent tissues. As shown in Figure 1, the expression of miR‐1301‐3p, miR‐1296‐5p, and miR‐532‐5p was decreased while the expression of miR‐455‐3p and miR‐551b‐3p was increased in PTC tissues compared to A‐benign tissues. miR‐1301‐3p was downregulated, and miR‐551b‐3p was upregulated in PTC tissues compared to benign nodules.

### 3.2 Aberrant miRNA expression due to cancerous cell alterations

**FIGURE 2:**
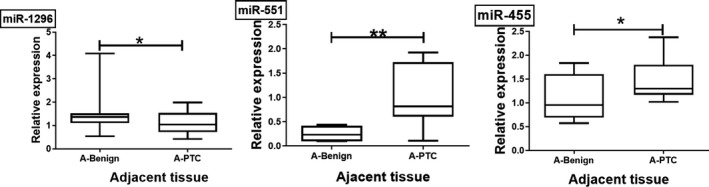
Aberrant miRNA expression due to cancerous cell alterations. Compared to the A‐benign group, miR‐1296‐5p was significantly downregulated and miR‐551b‐3p and miR‐455‐3p were upregulated in the A‐PTC group (*p* = 0.028, *p* = 0.002, *p* = 0.004, respectively)

As shown in Figure 2, miR‐1296‐5p was significantly downregulated and miR‐551b‐3p and miR‐455‐3p were upregulated in the A‐PTC group compared to the A‐benign group (*p* = 0.028, *p* = 0.002, and *p* = 0.004, respectively).

### 3.3 The correlation between miRNA expression and clinicopathological features

The miR‐1296‐5p expression was lower in large tumors, multiple foci, and advanced TNM stages. miR‐455‐3p expression was higher with advanced TNM stage, large tumors, and younger age. miR‐532‐5p was lower with advanced age, TNM stage, and lymph node metastasis. These pairs are shown in Table 2 along with their Kendall‐correlation coefficient values. We also analyzed miRNAs and other clinical features, such as ATA classification, tumor subtype, and Tg expression, but no significant difference was found. The details are shown in Tables S2–1, 2, 3, and 4.

**TABLE 2:** Analysis of miRNAs expression and clinic‐pathological features

Parameters		miR‐1296‐5p			miR‐455‐3p			miR‐532‐5p		
		Expression	*p*	*r*	Expression	*p*	*r*	Expression	*p*	*r*
TNM staging	I	0.35 (0.69–0.11)	0.028**	0.185	1.87 (4.25–1.36)	0.004**	0.22	0.64 (1.57–0.17)	0.013*	0.173
	Ⅱ﹣Ⅳ	0.16 (0.30–0.09)			5.14 (8.67–3.08)			0.32 (0.48–0.22)		
Tumor Size	≥2 cm	0.10 (0.34–0.06)	0.006**	0.190	2.09 (8.09–1.45)	0.039*	0.180			
	<2 cm	0.39 (0.79–0.24)			3.51 (8.86–1.80)					
Number of foci	Multi	0.30 (0.36–0.08)	0.047*	0.156						
	Single	0.42 (0.80–0.09)								
Age	≥45				4.60 (8.86–2.30)	0.028*	0.177	0.36 (0.58–0.21)	0.045*	0.191
	<45				1.77 (9.41–1.21)			0.67 (1.82–0.21)		
Lymph node metastasis	Yes							0.55 (1.57–0.35)	0.013*	0.170
	No							0.22 (0.75–0.13)		

Note

Kendall correlation coefcients and its adjusted *p*‐value are listed.

### 3.4 Analysis of the diagnostic value of miRNAs

**FIGURE 3:**
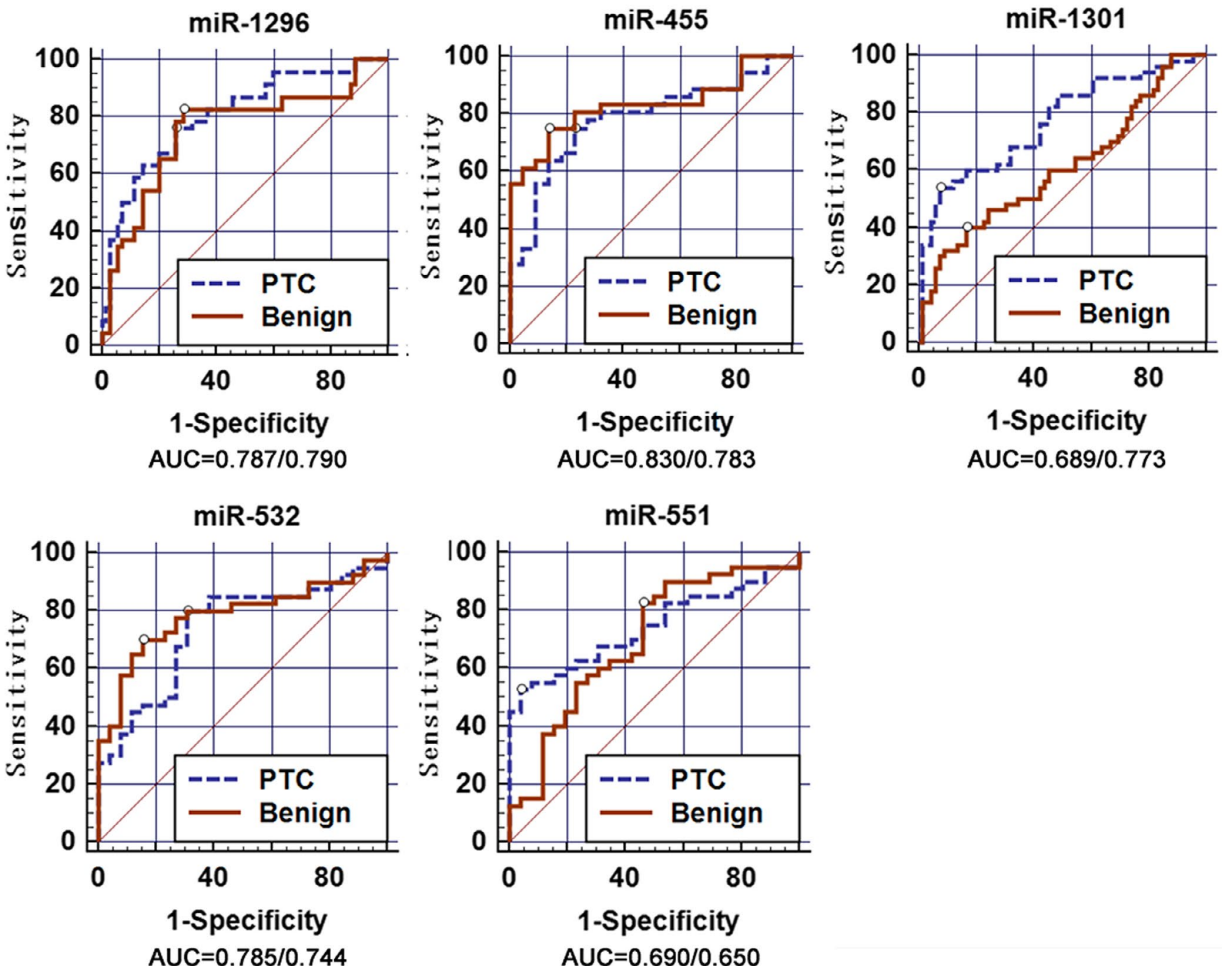
Analysis of the diagnostic value of miRNAs. ROC curves used to evaluate the diagnostic value of the five miRNAs. The AUCs for miR‐1301‐3p, miR‐1296‐5p, miR‐455‐3p, miR‐532‐5p, and miR‐551b‐3p were 0.773, 0.790, 0.783, 0.744, and 0.650 in PTC vs A‐benign, respectively. miR‐1295‐5p exhibited a better diagnostic value, with 80.6% and 71.4% sensitivity and specificity, respectively. (AUC = Benign/PTC)

**FIGURE 4:**
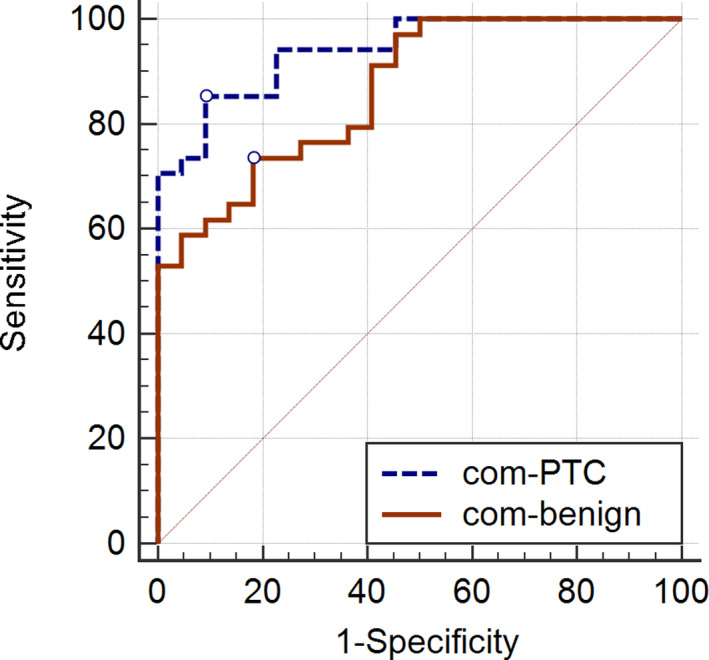
The diagnostic value of the combination of five miRNAs. The AUC for the combination of miR‐1301‐3p, miR‐1296‐5p, miR‐455‐3p, miR‐532‐5p, and miR‐551b‐3p was 0.941 in PTC vs A‐benign, with 82.14% and 100% sensitivity and specificity, respectively. The AUC was 0.866 in benign vs. A‐benign lesions, with 75.00% and 81.82% sensitivity and specificity, respectively

To evaluate the diagnostic values of miRNAs in distinguishing normal tissue from benign thyroid nodular goiter or PTC tissues, we plotted ROC curves. The results are presented in Figures 3 and 4. Using the area under the curve (AUC) to assess diagnostic value, the optimal −ΔCT cut‐off values for miR‐1301‐3p, miR‐1296‐5p, miR‐455‐3p, miR‐532‐5p, and miR‐551b‐3p to distinguish cancerous cells from normal tissues were −12.96, −10.45, −7.90, −7.69, and −10.96, respectively. The AUCs for miR‐1301‐3p, miR‐1296‐5p, miR‐455‐3p, miR‐532‐5p, and miR‐551b‐3p were 0.773, 0.790, 0.783, 0.744, and 0.650, respectively. The combination of these five miRNAs demonstrated the best prediction accuracy to distinguish cancerous cells from normal tissues with an average AUC of 0.941, with 82.14% and 100% sensitivity and specificity, respectively. The details are shown in Table 3.

**TABLE 3:** Receiver operating characteristic (ROC) details

miRNAs	Tissue	Sensitivity	Specificity	AUC	*p*‐value	Cut‐off value (−ΔCT)	95% CI
miR‐1296‐5p	A‐benign *vs* benign	80.65	73.53	0.787	<0.0001	−10.35	0.721–0.865
	A‐benign vs PTC	80.60	71.4	0.790	<0.0001	−10.45	0.731–0.875
miR‐455‐3p	A‐benign *vs* benign	67.86	85.00	0.830	0.0003	−8.43	0.614–0.871
	A‐benign *vs* PTC	80.00	65.00	0.783	0.0002	−7.82	0.617–0.834
miR‐1301‐3p	A‐benign *vs* benign	48.15	93.22	0.689	<0.0001	−12.20	0.639–0.810
	A‐benign *vs* PTC	53.57	93.22	0.773	<0.0001	−12.96	0.714–0.868
miR‐532‐5p	A‐benign *vs* benign	70.00	84.62	0.785	<0.0001	−8.50	0.666–0.876
	A‐benign *vs* PTC	77.55	69.23	0.744	0.0004	−7.69	0.621–0.831
miR‐551b‐3p	A‐benign *vs* benign	82.50	53.85	0.690	0.0040	−12.81	0.571–0.803
	A‐benign *vs* PTC	56.90	96.15	0.650	<0.0001	−10.96	0.621–0.820
Combination	A‐benign *vs* benign	75.00	81.82	0.866	<0.0001	0.47	0.749–0.942
	A‐benign *vs* PTC	82.14	100.00	0.941	<0.0001	0.77	0.896–0.993

## 4 DISCUSSION

In this study, we selected five miRNAs and determined their expression in PTC tumors to improve our understanding of the pathogenesis of PTC and identify potential biomarkers for PTC. We found that three miRNAs were downregulated and that two miRNAs were upregulated in PTC tumor tissues. Through ROC curve analysis, we speculated that miR‐1301‐3p, miR‐1296‐5p, miR‐455‐3p, miR‐532‐5p, and miR‐551b‐3p may serve as potential biomarkers for the diagnosis of PTC.

Because of the substantial proportion of indeterminate lesions induced with this tumor, scholars have turned to discover diagnostic biomarkers to improve PTC diagnostic efficiency^5^ and evaluate tumor biological behavior and manage operations.^6^ miRNAs have been regarded as such biomarkers since their expression levels reflect the occurrence and progression of disease.

Recent studies have shown that the above five miRNAs are closely related to the pathogenesis of malignant tumors and play different roles in different tumors. miR‐1301 is regarded as an anti‐oncogene in liver cancer but as an oncogene in prostate cancer.^7^, ^8^, ^9^ miR‐1296 was demonstrated to be a tumor suppressor gene in hepatocellular carcinoma and breast cancer^10^ but acted as a carcinogenic gene in colorectal cancer.^11^ miR‐455‐3p and miR‐532‐5p are associated with breast cancer, gastric cancer, pancreatic cancer, colon cancer, and nonsmall cell‐lung cancer.^12^, ^13^ Regarding thyroid cancer, miR‐1301‐3p was decreased in PTC tissues and suppressed thyroid cancer cell proliferation through the ABHD11‐AS1‐miR‐1301‐3p/STAT3 feedback loop based on J. Wen's study.^14^ For miR‐455‐3p, miR‐532‐5p, and miR‐551b‐3p, a study indicated that their expression was downregulated and that miR‐551b‐3p expression was upregulated in PTC tissues.^15^ Our results were similar to those of the above studies. However, we obtained opposite results for miR‐455‐3p. This difference may be due to regional and ethnic differences in patients, which needs to be further verified with a larger sample size. Regardless of this, we still believe that these five miRNAs may serve as potential diagnostic biomarkers for PTC.

To verify that the alterations in miRNAs levels were the result of alterations in cancerous cells, Peng et al. found that the expression of miR‐30a‐3p in nontumor tissues adjacent to PTC was lower than that in non‐nodular tissue adjacent to benign thyroid nodular goiter.^16^ Interestingly, we obtained similar results. This suggests that changes in miRNA expression may originate through the effects of the tumor and not because of individual factors. Whether this effect is associated with immune cell function or the influence of cancerous cells will be the subject of future studies. In summary, our study is in accordance with previous findings that abnormal miRNA expression occurs in the whole thyroid regardless of whether it is an early genetic event in PTC carcinogenesis or carcinogenic miRNA changes.^17^, ^18^ Moreover, aberrant miRNA expression in A‐PTC compared to A‐benign PTC may overcome the limitations of ultrasound‐guided fine needle aspiration, which is the mainstay of thyroid disorders, but does not accurately categorize 20% of thyroid lesions due to the limitations of operator subjectivity and expertise and the small sample size.^19^ These miRNAs, as tumor‐associated changes, might be detectable even in a small sample size or difficult position.

More recent studies have demonstrated a noticeable association between miRNA expression profiles and other clinicopathological features, such as tumor size, lymph metastasis, TNM stage, and PTC histological subtypes.^20^ Wang J et al^12^ recently reported that miRNA‐455‐3p acted as an anti‐oncogene in colon cancer, which was correlated with cell proliferation and apoptosis. Previous studies have shown that miR‐1296 plays an anti‐oncogenic role in prostate cancer and breast cancer. Several studies have reported that miR‐532‐5p participates in tumor progression.^13^ In our study, the results showed that miRNA expression is correlated with clinicopathologic parameters in PTC tumors and that the five miRNAs with altered expression may serve as prognostic biomarkers for PTC tumors. Further study of the molecular mechanism of these miRNAs involved in tumor metastasis and invasion will benefit the prognosis of PTC.

The ROC curve is extensively used to estimate the diagnostic accuracy of molecules. By plotting the ROC curves, He et al. proposed that miRNAs could be potential molecules to distinguish PTC from normal tissues in an article published in 2005.^17^ They found that diagnosis with a single miRNA was not satisfactory; then, as expected, the accuracy was improved when two or more miRNAs were used for diagnosis. He and his colleagues were able to blindly delineate the cancer status in 12 tumor samples with 100% accuracy by combining five miRNAs.^18^ Nikiforova^21^ found the combined use of two or more miRNAs to diagnose PTC, with a sensitivity of 100%, specificity 94%, and accuracy 95%. Similar to the above study, we found that the five miRNAs had a diagnostic value for thyroid cancer. In particular, the diagnostic value is somewhat different from what we reported previously because the sample size included is somewhat different.^22^ Although the AUC of a single miRNA was unsatisfactory, we developed a 5‐miRNA signature of miR‐1301‐3p, miR‐1296‐5p, miR‐455‐3p, miR‐532‐5p, and miR‐551b‐3p with excellent diagnostic value in PTC patients.

It must be noted that our study presents some limitations. First, due to sample size restrictions, we did not investigate the specific functions of the miRNAs to distinguish benign from malignant tissues, which is very important for a diagnostic biomarker. However, several studies have reported that many miRNAs may act as diagnostic biomarkers for thyroid cancer.^23^ We believe that the newly discovered miRNAs specifically expressed in PTC will provide new and useful information for future researchers. Second, our study was not performed on FNAC samples, which are needed before surgical intervention, especially in indeterminate samples. There are still no universally accepted reference miRNAs that could be used as normalizers for miRNA quantification in FNAC preparations.^24^ Then, we can further select and validate potential miRNAs from the five aberrantly expressed miRNAs. Finally, a multicenter study including a large number of clinical cases, particularly indeterminate lesions, is needed.

## 5 CONCLUSION

Taken together, our results indicated that miR‐1296‐5p, miR‐1301‐3p, miR‐532‐5p, miR‐551b‐3p, and miR‐455‐3p are aberrantly expressed in papillary thyroid carcinomas and correlated with clinicopathological features. ROC curve analysis indicated that the five miRNAs have diagnostic value. Therefore, we speculate that these five altered miRNAs may serve as potential diagnostic and prognostic biomarkers for PTC.

## CONFLICT OF INTEREST

The authors declare that they have no competing interests.

## AUTHOR CONTRIBUTIONS

Xiang‐yu Zhou and De‐hui Qiao designed and performed all experiments, analyzed the data, and wrote the manuscript. Xue‐mei He and Xian Deng involved in data analysis/interpretation. Yi‐chi Ji and Hui Yang involved in statistical analysis. Cheng lian involved in data acquisition.

## Supporting information

Table S1‐2

## Data Availability

The data that support the findings of this study are available from the corresponding author upon reasonable request.
