# Supplementary material for: Aberrant expression of five miRNAs in papillary thyroid carcinomas
Source: J Clin Lab Anal. 2021 Jul 16;35(9):e23907. doi: 10.1002/jcla.23907 (PMC8418488; doi:10.1002/jcla.23907)
Supplement: Supplementary file 1 — Table S1‐2 [file JCLA-35-e23907-s001.docx]

**Supplementary Table I.**

Overview of studies using miRNA microarray and bioinformatics analysis to compare PTC and control

| miRNAs | study | Validation samples | | Expressing in PTC |
| --- | --- | --- | --- | --- |
| miR-1301-3p | Wen J, et al. (2019)[14] | Fresh frozen, 82 PTC vs. 82 non‐tumor | Underexpressed | |
| miR-1296-5p | Oncomir (http://www.oncomir.org/) | none | Underexpressed | |
| miR-532-5p | Swierniak M, et al. (2013)[15] | Fresh frozen, 14 PTC vs. 14 adjacent normal thyroid | Underexpressed | |
| miR-551b-3p | Swierniak M, et al. (2013)[15] | Fresh frozen, 14 PTC vs. 14 adjacent normal thyroid | Overexpressed | |
| miR-455-3p | Swierniak M, et al. (2013)[15] | Fresh frozen, 14 PTC vs. 14 adjacent normal thyroid | Underexpressed | |

**Supplementary Table II.** **Analysis of miRNAs expression and clinic-pathological features**

**Table II-1**

| Parameters | miR-1301-3p | miR-1296-5p |
| --- | --- | --- |

|  | | Expression | *P* | Expression | *P* |
| --- | --- | --- | --- | --- | --- |
| Age | **≥45** | 0.28(0.69-0.14) 0.425 | | 0.34(0.6-0.09) 0.963 | |
|  | <**45** | 0.57 (1.24-0.11) | | 0.31(0.51-0.08) | |
| Sex | **Female** | 0.22(0.89-0.13) 0.528 | | 0.31(0.49-0.10) 0.726 | |
|  | **Male** | 0.46 (0.90-0.15) | | 0.45(0.88-0.07) | |
| Tumor size | **≥2cm** | 0.19(0.64-0.12) 0.165 | | 0.10(0.34-0.06) **0.006**** | |
|  | <**2cm** | 0.56 (0.88-0.17) | | 0.39(0.79-0.24) | |
| Number of foci | **Multi** | 0.22(0.93-0.15) 0.743 | | 0.30(0.36-0.08)  **0.047*** | |
|  | **Single** | 0.58 (0.91-0.12) | | 0.42(0.80-0.09) | |

The expression values were calculated by 2^−ΔΔCT^ and the medians and interquartile were presented;

*p* value was calculated using the Mann-Whitney U test. (**P*<0.05, ***P*<0.01).

| Parameters | | miR-1301-3p | | miR-1296-5p | |
| --- | --- | --- | --- | --- | --- |
|  |  | **Expression** | ***P*** | **Expression** | ***P*** |
| ASA classification | **Low** | 0.45 (0.93-0.13) 0.248 | | 0.35(0.78-0.08) 0.067 | |
|  | **Mid-High** | 0.22 (0.84-0.14) | | 0.18(0.33-0.10) | |
| TNM staging | **I** | 0.62 (1.24-0.12) 0.143 | | 0.35(0.69-0.11) **0.028**** | |
|  | **Ⅱ**﹣**Ⅳ** | 0.18 (0.50-0.14) | | 0.16(0.30-0.09) | |
| Tumor subtype | **PTC** | 0.25 (0.77-0.14) 0.112 | | 0.58(0.84-0.29) 0.259 | |
|  | **FVPTC** | 1.21 (2.42-0.62) | | 0.27(0.44-0.10) | |
| Tg | <**77ng/ml** | 0.55 (0.82-0.18) 0.700 | | 0.26(0.39-0.10) 0.474 | |
|  | **≥77ng/ml** | 0.28 (0.94-0.13) | | 0.14(0.42-0.07) | |
| Extra thyroidal extension | **Yes** | 0.19(1.02-0.13) 0.884 | | 0.14(0.46-0.10) 0.081 | |
|  | **No** | 0.43(0.79-0.14) | | 0.33(0.88-0.18) | |

**Table II-2**

The expression values were calculated by 2^−ΔΔCT^ and the medians and interquartile were presented;

*p* value was calculated using the Mann-Whitney U test. (**P*<0.05, ***P*<0.01).

**Table II-3**

| Parameters | | miR-455-3p | | miR-551-3p | | miR-532-5p | |
| --- | --- | --- | --- | --- | --- | --- | --- |
|  |  | **Expression** | ***P*** | **Expression** | ***P*** | **Expression** | ***P*** |
| Age | **≥45** | 4.60(8.86-2.30) **0.028*** | | 11.09(17.59-1.01) 0.184 | | 0.36(0.58-0.21) **0.045*** | |
|  | <**45** | 1.77(9.41-1.21) | | 3.26(9.23-1.40) | | 0.67(1.82-0.21) | |
| Sex | **Female** | 3.26(9.74-1.48) 0.638 | | 10.69(15.60-2.77) 0.460 | | 0.48(0.86-0.15) 0.126 | |
|  | **Male** | 2.22(4.19-1.80) | | 7.60(13.45-2.13) | | 0.60(2.84-0.24) | |
| Tumor size | **≥2cm** | 2.09(8.09-1.45)  **0.039*** | | 4.47(16.81-0.78) 0.416 | | 0.36(0.64-0.21) 0.169 | |
|  | <**2cm** | 3.51(8.86-1.80) | | 5.89(13.67-1.90) | | 0.57(1.82-0.24) | |
| Number of foci | **Multi** | 1.94(5.38-1.47) 0.104 | | 7.34(12.24-1.09) 0.950 | | 0.55(1.75-0.36) 0.056 | |
|  | **Single** | 2.76(11.46-1.86) | | 4.76(19.27-1.15) | | 0.44(1.33-0.13) | |
| Lymph node metastasis | **Yes** | 2.27(5.81-1.48) 0.102 | | 3.86(10.12-0.05) 0.202 | | 0.55(1.57-0.35) **0.013*** | |
|  | **No** | 3.18(16.53-1.80) | | 6.77(15.44-1.25) | | 0.22(0.75-0.13) | |

The expression values were calculated by 2^−ΔΔCT^ and the medians and interquartile were presented; p value was calculated

using the Mann-Whitney U test. (**P*<0.05,***P*<0.01).

**Table II-4**

| **Parameters** | | miR-455-3p | | miR-551-3p | | miR-532-5p | |
| --- | --- | --- | --- | --- | --- | --- | --- |
|  |  | **Expression** | ***P*** | **Expression** | ***P*** | **Expression** | ***P*** |
| ASA classification | **Low** | 2.73(9.57-1.48) 1.000 | | 7.60(14.42-1.95) 0.290 | | 0.50(1.37-0.15) 0.631 | |
|  | **Mid-High** | 2.43(5.38-1.80) | | 2.24(14.63-0.73) | | 0.51(1.12-0.38) | |
| TNM staging | **I** | 1.87(4.25-1.36) **0.004**** | | 4.41(12.78-1.16) 0.310 | | 0.64(1.57-0.17) **0.013*** | |
|  | **Ⅱ**﹣**Ⅳ** | 5.14(8.67-3.08) | | 9.05(17.35-1.09) | | 0.32(0.48-0.22) | |
| Tumor subtype | **PTC** | 2.88(8.86-1.69) 0.457 | | 5.15(13.45-1.52) 0.652 | | 0.46(1.12-0.21) 0.443 | |
|  | **FVPTC** | 1.97(15.55-1.10) | | 34.99(79.60-0.54) | | 0.99(1.48-0.42) | |
| Tg | <**77ng/ml** | 2.09(6.36-1.49) 0.332 | | 6.76(12.78-2.21) 0.864 | | 0.47(1.24-0.15) 0.901 | |
|  | **≥77ng/ml** | 4.60(9.15-1.70) | | 2.39(17.92-0.93) | | 0.51(1.37-0.24) | |
| Extra thyroidal extension | **Yes** | 1.99(5.38-1.67) 0.525 | | 2.24(9.63-0.99) 0.203 | | 0.51(1.92-0.31) 0.650 | |
|  | **No** | 2.76(9.04-1.59) | | 7.60(16.31-1.53) | | 0.48(1.29-0.15) | |

The expression values were calculated by 2^−ΔΔCT^ and the medians and interquartile were presented; p value was calculated

using the Mann-Whitney U test. (**P*<0.05,***P*<0.01).
